# Challenges in Respiratory Medicine The need for Integrated Tuberculosis and Respiratory care in low-resource settings

**DOI:** 10.1136/thorax-2024-222170

**Published:** 2025-08-28

**Authors:** Jacqueline Wanjiku Kagima, Obianuju B. Ozoh, Stellah Mpagama, Nora Engel, Maia Lesosky, Jason Madan, Jeremiah Chakaya, Jamilah Meghji

**Affiliations:** 1 Kenyatta National Hospital, Nairobi, Kenya; 2 Centre for Respiratory Diseases Research, Kenya Medical Research Institute, Nairobi, Kenya; 3 Department of Medicine, College of Medicine, University of Lagos, Lagos, Nigeria; 4 Lagos University Teaching Hospital, Lagos, Nigeria; 5 Kibongo’to Infectious Diseases Hospital, Kilimanjaro, Tanzania; 6 The Athena Institute, Vrije University Amsterdam, Amsterdam, The Netherlands; 7 National Heart and Lung Institute, Imperial College London, London, UK; 8 Warwick Medical School, University of Warwick, UK

**Keywords:** Tuberculosis, Post-tuberculosis, Chronic respiratory diseases, Integration, Health systems

## Abstract

Pathways to care for people with chronic respiratory diseases (CRDs) remain inadequate in many low- and middle-income countries (LMICs). In this opinion piece we describe opportunities for the integration of tuberculosis (TB) and respiratory care, as a means of improving patient outcomes.

TB and CRDs are intricately linked in many LMICs. People with pulmonary TB (PTB) and CRDs experience similar symptoms, including breathlessness, cough, and chest pain. They may have similar risk factors for disease, including smoking and occupational exposures. PTB is a direct cause of lung damage in the form of post-TB lung disease. Both groups are often directed to TB services as a first point of contact, where they are known as ‘people with presumptive TB’. However, despite the overlap in risk factors, symptoms, and harms described above, public health and clinical care pathways for TB and CRDs remain almost entirely separate in many LMICs.

It is likely that co-ordinated approaches to prevention, diagnosis and care would improve patient outcomes. Strategies may include upstream public health interventions to address shared risk factors, the use of shared diagnostic pathways, the provision of decentralised access to both TB and CRD care, and co-ordinated information provision about the risk factors and symptoms of both conditions. Health-related benefits may include more timely diagnosis of CRDs, improved CRD treatment and care, and reduced inappropriate empirical TB treatment or retreatment. Integrated approach may be particularly timely, given increasing scarcity in global health donor funding.

However, we also highlight the need for further data in this space. Robust approaches to the design and evaluation of integrated services are lacking. Observational work describing the core components and potential impact of integrated care, and pilot models of integrated care are much needed to inform person-centred approaches to TB and respiratory care in LMICs.

## Background

Globally, more than 10 million people develop Tuberculosis disease (TB) every year, a quarter of whom live in Africa. TB disease is known to have a profound impact on those affected, as well as their households and families, with many incurring catastrophic direct and indirect costs related to health seeking or loss of income and employment.[1] Mortality remains high, and TB was the world’s leading cause of death from a single infectious agent with an estimated 1.25 million deaths in 2023.[2] Amongst those who survive TB disease, many incur long-term sequelae following treatment completion, including residual lung impairment.

Alongside this high burden of TB disease, there is a rising burden of non-communicable diseases in low- and middle income countries (LMICs), including chronic respiratory diseases (CRDs).[3] There are limited primary data with which to estimate the prevalence of CRDs,[4] but poverty-related risk factors for CRDs are widespread in LMICs and include indoor and outdoor air pollution, in-utero and early childhood undernutrition, and early respiratory tract infections which may impair lung development. Adolescents and adults may also be exposed to tobacco smoke and vaping, air pollution, respiratory infections, as well as occupational exposures, which together may lead to lung damage, exacerbations of existing lung disease and accelerated decline in lung function. There is a growing body of data showing a high burden of small lungs, asthma, and COPD in LMICs in relation to these exposures.

In our experience, largely rooted in the African context, TB disease and CRDs are intricately linked from a patient experience, public health and clinical perspective. However, integrated approaches to their prevention, diagnosis and care remain lacking.

In this manuscript we discuss the need for and challenges around integrating TB and respiratory care in low-resource settings. Our review reflects the perspectives of the authors – no formal literature search has been completed.

### Intersection between TB and CRDs

CRDs and TB have shared risk factors. Tobacco smoke is associated with increased risk of TB infection, TB disease, and TB mortality and is a direct cause of COPD.[5] Occupational exposures such as silica are associated with increased risk of TB disease and lead to progressive and irreversible interstitial lung disease. People living with HIV have an increased risk of incident TB disease, even in the context of viral suppression, and may face an increased burden of chronic airway obstruction.[6] Perhaps most importantly, both CRDs and TB are associated with poverty and deprivation, and upstream determinants such as poor housing, overcrowding, use of dirty fuels, and malnutrition are core drivers of both poor lung health and TB exposure and disease.

People with pulmonary TB (PTB) and CRDs also experience similar symptoms, including breathlessness, cough, and chest pain. In LMICs people presenting with respiratory symptoms are often directed to TB services as a first point of contact, and are known as ‘people with presumptive TB’ (PPTB). However, a recent systematic review including seven studies (n=4429 participants) in sub-Saharan Africa showed that only half of symptomatic adults presenting to TB services for investigation were diagnosed with TB disease.[7] Alternative diagnoses amongst those not diagnosed with TB are poorly described, but data from The Gambia and Zimbabwe suggest these include non-TB respiratory infections, CRDs, malignancy, and heart failure.[8, 9]

PTB may also directly cause respiratory pathology, with a high burden of residual post-TB lung disease (PTLD) described amongst TB survivors in LMICs. Up to half of those successfully treated for PTB disease are left with residual structural or functional lung impairment. Many of these individuals experience ongoing respiratory symptoms, exacerbations, or functional impairment, with associated health seeking and loss of income and employment. Empirical TB-retreatment as a result of ongoing symptoms has been described in this group, [10] and post-TB respiratory morbidity and mortality accounts for almost half of the disability adjusted life years (DALYs) lost from TB disease annually.[11] People who have successfully completed treatment for pulmonary tuberculosis also experience an increased mortality rate, compared with the general population (estimated standardised mortality rate 2.9 (95% CI 2.2-3.8)).[12] Cause of death data are limited, but suggest that this may be driven by cancers, cardiovascular disease, and respiratory disease. [12]

### The risk of compounded harm

Given the relationships described above, the clinical, economic and social harms related to TB disease, TB treatment and CRDs may be overlapping, and likely compound each other.

Many patients may experience a combination of acute illness related to TB disease, lung damage related to pre-existent respiratory pathology, and direct respiratory damage caused by TB disease. Symptomatic individuals with missed diagnoses of CRDs may receive recurrent courses of antibiotics or empirical TB re-treatment, compounding challenges around antimicrobial resistance, and exposing them to un-necessary TB drug adverse effects and toxicity. Failure to accurately diagnose CRDs may mean that early therapeutic interventions which could improve quality of life, physiology and function, and prevent disease progression are delayed or never accessed.

Additionally, the financial impact of TB and CRDs may be significant when experienced together. Although TB treatment is provided free of charge, the direct and indirect costs incurred during disease and treatment are well described, with a high proportion of TB affected households facing catastrophic costs (>20% of annual household income),[13] or dissaving (use of savings, selling of assets, or borrowing of money).[14] These costs are associated with worsening poverty, the withdrawal of children from school, and with poor TB treatment outcomes.[15] The financial impact of CRDs on patients in LMICs is less well described, but may be significant: CRDs may cause functional impairment and exacerbations, resulting in time off work and loss of income, and the high frequency of health seeking observed for chronic respiratory symptoms in both the public and private sector may carry significant cost.[16] There are likely significant health system costs associated with recurrent health seeking for undiagnosed respiratory symptoms, avoidable admissions for those with untreated CRDs, and inappropriate TB treatment for those who are misdiagnosed.

In addition, both TB and CRD affected communities may face stigma as a result of chronic cough.[17] This is likely to have worsened with the recent Covid-19 pandemic. Stigma around TB disease itself is well described in the literature, and has served as both a barrier to diagnosis and treatment completion. Chronic conditions such as CRDs are also stigmatised in many settings, providing a further barrier to CRD diagnosis and the uptake of respiratory treatments such as inhalers.[18]

### Fragmentation of existing health systems

Despite the overlap in risk factors, symptoms, and harms described above, public health and clinical care pathways for TB and respiratory disease remain almost entirely separate in many LMICs.

TB services have historically benefited from strong donor support, and are delivered by national TB programmes (NTPs), with clear governance and reporting pathways, trained staff, secure procurement of diagnostic tests and medication, and ring-fenced funding. In contrast, respiratory care often sits within broader health services, often with less well supported diagnostic pathways, and significant staff constraints. This limits access to investigation, diagnosis, treatment and follow up. In many sites the staff delivering TB and CRD care come from different clinical backgrounds and training pathways, often with limited communication between groups.

Respiratory exposures are not routinely addressed within TB services. For example, although brief counselling for smoking cessation is a low-cost behavioural intervention recommended by the WHO, the integration of smoking cessation services within many NTPs has been limited. A review of smoking cessation service provision in South-East Asia described limited integration of TB and tobacco control activities at the policy, planning, implementation and monitoring levels within health services as a major barrier to implementation.[19]

In many LMICs, the first investigation for those presenting with chronic cough is either sputum smear or XPert MTB/Rif (Cephaid, Sunnyvale, USA). Although investigations such as chest-radiographs are increasingly used in both active and passive TB case finding, digital reading algorithms remain focused on TB disease and use of imaging for broader respiratory diagnoses is limited or absent.[20]

Similarly, linkage to respiratory care for those with residual post-TB respiratory symptoms is limited. Several countries in Africa and Latin America are increasingly adopting recommendations for the diagnosis and management of post-TB lung disease within TB programme (E.g. through Pulmonary rehabilitation) to reduce symptoms and improve functional capacity, but implementation has been limited to date.

### Opportunities for integrated prevention and care

**Figure 1:**
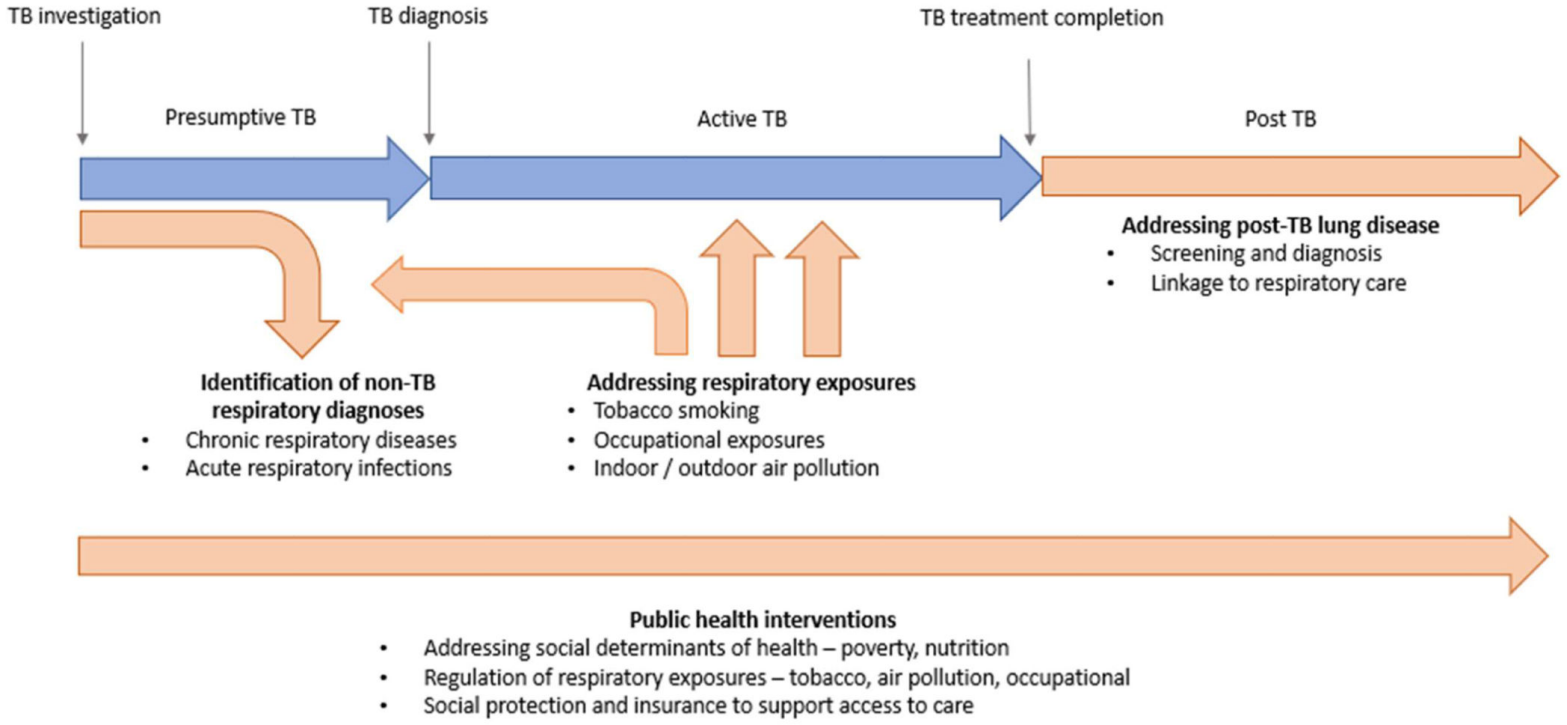
Framework for the integration of TB and respiratory care across the TB care cascade Blue: existing TB care cascade; Orange: Proposed interventions to deliver integrated respiratory care

Going forward – there is an opportunity to integrate pathways for the provision of TB and CRD services within health systems, bringing together TB and CRD prevention, diagnosis and care (Figure 1). A co-ordinated approach to care may be particularly timely, given recent shifts and increasing scarcity in global health donor funding. Integrated interventions may include upstream public health interventions to prevent both TB and respiratory disease, shared diagnostic pathways for these conditions, combined information provision about the risk factors and symptoms of both conditions, and integrated clinical care services with access to both TB and CRD care for patient groups. Potential health-related benefits may include more timely diagnosis of CRDs, improved treatment and follow up for CRD care, and reduced inappropriate empirical TB treatment or retreatment.

However, significant questions remain about how this might be achieved. It may be most efficient to leverage the strong backbone of TB services, which have been built over many decades, such that TB services are themselves able to deliver CRD diagnosis, respiratory counselling, and linkage to respiratory care. Alternatively, it may be that we need strengthened CRD diagnostic and care services running in parallel to TB pathways, with robust communication and referral pathways between these. Implementation data in this space is largely absent, and it is notable that previous attempts at integrated TB and CRD service delivery, such as the ‘Practical Approach to Lung Health’(PAL) model – an approach developed by the World Health Organisation to improve the quality of care of those presenting to primary care with respiratory symptoms – have had limited uptake.[21] Moreover, previous literature highlights that integration of services may not always have the intended benefit, or could have unintended harms.[22]

### Robust design and evaluation

Although the case for integration of TB and CRD services appears clear, robust approaches to the design and evaluation of integrated services are needed. Existing frameworks for the development and evaluation of complex health interventions may be particularly helpful to guide this approach.[23]

**Figure 2:**
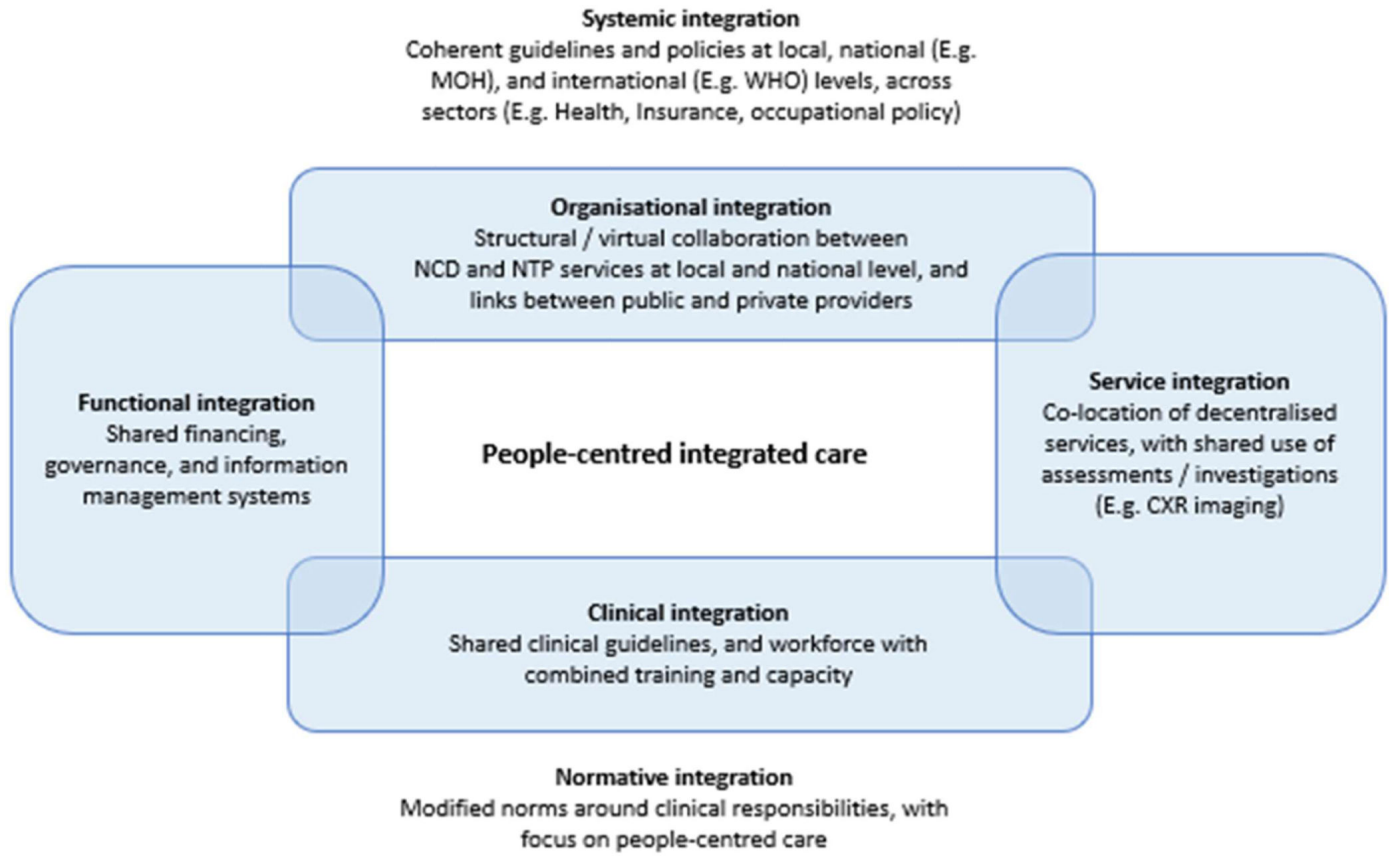
Proposed conceptual framework for people-centred integrated TB-respiratory care, adapted from Fulop and Molum[24] MOH – Ministry of health; WHO – World Health Organisation; NCD – Non communicable disease; NTP – National TB Programme; CXR – Chest radiograph

When designing integrated TB and CRD clinical services, it will be necessary to understand what aspects of clinical care could be brought together, and how health systems could achieve this in terms of staffing, financing, and diagnostic and information systems. The perspectives of patients, affected communities, and health care providers around the risks, benefits, and feasibility of these changes must be the starting point for programme design. We will need to explore the norms which would be required to make these changes sustainable and acceptable over time, and may need to consider how broader social sectors, including social security and insurance provision, education and occupational services might shape both respiratory health and access to care, and therefore the success of these integrated approaches (Figure 2).

Broad input from stakeholders will be needed to understand which patient, provider, and health system outcomes should be prioritised when evaluating the impact of integrated approaches to care. Evaluation approaches must identify benefits, but also unintended harms of integration – for example a focus on respiratory care may divert resources away from other non-communicable diseases. We will also need to understand the cost of these novel pathways of care, to determine whether they can be achieved using existing limited resources, or whether and where additional funding is required. People in many settings seek care from a wide array of public and private providers, and the delivery and impact of these changes on both sectors must be considered. We must also seek to understand what aspects of integrated care services do or don’t work, for whom, and why, if we are to ensure equitable improvements in health. Lastly, it will be critical to work with local, regional and international stakeholders who are involved in delivering, designing and funding care, to identify the data they would need to inform their decisions about implementation and scale up of integrated approaches.

### Next steps

Going forward, it is important to acknowledge that there is unlikely to be a ‘one size fits all’ model of integrated TB-CRD care for use across LMICs. Health systems will likely need to adapt to the burden of CRDs in their local setting, the existing context of care, and local funding pathways and constraints. However, in depth observational research to understand the core components and potential impact of integrated care, and rich data on pilot models of integrated care are much needed if we are to move towards person-centred approaches to TB and respiratory care in LMICs.
